# Patient-Centered, Theory-Based, Online Intervention to Promote a Physically Active Lifestyle for People With Multiple Sclerosis: Protocol for a Randomized Controlled Trial

**DOI:** 10.2196/66091

**Published:** 2025-07-17

**Authors:** Chungyi Chiu, Vaishnavi Sridharan, Mojtaba Khaliji, Md Refat Uz Zaman Sajib, Xiaotian Gao, Kathleen Zackowski

**Affiliations:** 1 Department of Health and Kinesiology University of Illinois Urbana-Champaign Urbana, IL United States; 2 Baylor College of Medicine Houston, TX United States; 3 National Multiple Sclerosis Society New York, NY United States

**Keywords:** self-management, telerehabilitation, physical activity, active lifestyle, multiple sclerosis

## Abstract

**Background:**

People with multiple sclerosis (MS) experience life challenges due to the nature of disease progression. Although an active lifestyle has been shown effective for symptom and relapse management, most people with MS lead a sedentary lifestyle and do not reach the recommended physical activity (PA) guidelines.

**Objective:**

This study aims to test the preliminary efficacy of a codeveloped, online self-management intervention based on the health action process approach (HAPA) adapted for people with MS to cultivate a physically active lifestyle.

**Methods:**

The 8-week intervention program was developed using community-based participatory research based on the HAPA. The program includes increasing awareness of incorporating PA in a daily routine, the benefits of physical activities for people with MS, developing motivation, utilizing social and environmental support, setting measurable goals and doable plans, and coping with barriers for long-term adoption of new behaviors. Community members with MS serve as peer coaches. We aimed to recruit 60 people with MS, randomizing them to an intervention group or a wait-list control group. Control group participants do not receive any intervention nor information for the period of study. Participants will complete PA measures (accelerometer and survey) and psychological assessments at baseline; after an 8-week intervention; and 3 months, 6 months, and 12 months postintervention. We hypothesize that the intervention group will have (1) higher scores on the HAPA questionnaires and (2) higher frequency, duration, and intensity of engagement in PA after the intervention than the control group. We will conduct descriptive analyses (means, SDs), chi-squared tests, independent *t* tests, paired *t* tests, repeated measures ANOVA, and 2-way (any 2 factors of conditions, disability severity levels, and time) and 3-way (conditions × disability severity levels × time) mixed model ANOVAs.

**Results:**

The study was funded in April 2018 and was delayed due to the COVID-19 pandemic. We invited peer coaches to review the accessibility of online modules in fall 2022. We finalized the module accessibility and trained peer coaches on how to use the online modules in December 2022. Since February 2023, we have recruited 50 participants to the trial who have been assigned to the intervention group (n=25) or control group (n=25). During the follow-up stage, 3 participants dropped out of the intervention group, and 4 participants dropped out of the control group. Enrollment ended in 2024, and data collection is expected to conclude by December 2025, with results anticipated to be published in January 2026.

**Conclusions:**

This study will test the effectiveness of using an evidence-based online intervention for self-management of physical activity behavior by people with MS. The results of the study will provide us with insightful information for designing community-based participatory research and implementing telerehabilitation interventions for people with MS.

**Trial Registration:**

ClinicalTrials.gov NCT05124522; https://clinicaltrials.gov/study/NCT05124522

**International Registered Report Identifier (IRRID):**

DERR1-10.2196/66091

## Introduction

### Background

Multiple sclerosis (MS) is a progressive neurodegenerative disorder that affects nearly 1 million Americans; this number has doubled since 1976 [1,2]. This underscores a pressing need for disease management and prevention efforts [2]. The growing number of cases is particularly concerning, as there exists a series of unmet needs in managing the unpredictable and evolving symptoms of MS. Approximately 80% to 90% of people with MS experience acute episodes of relapses, often resulting in partial or complete recovery [3]. However, it is important to note that recovery from these relapses is typically incomplete over time, leading to a substantial accumulation of neurological disability [3]. Common symptoms of MS encompass fatigue, spasms, difficulties with mobility and balance, cognitive and emotional alterations, and depression [4,5]. These symptoms can lead to a sedentary lifestyle, diminish physical and mental well-being, and subsequently result in physical and mental disabilities, ultimately reducing community participation [6].

People with MS have reported exacerbation of their symptoms when living a sedentary lifestyle [6]. Such a sedentary lifestyle is often associated with an elevated risk of developing comorbid conditions, including cardiovascular diseases, type 2 diabetes, obesity, various cancers, depression, hypertension, and stroke [6,7]. Compelling evidence suggests that, even in the case of a progressive form of the disease, engaging in physical activity (PA) offers numerous benefits, specifically alleviating MS symptoms, managing comorbidities, and reducing the frequency of relapses [8,9]. This, in turn, leads to improved quality of life and reduced health care costs, which are particularly crucial for people with MS [9,10]. Most significantly, PA effectively mitigates symptoms and slows the progression of MS [6,11-15].

The evidence-informed PA guidelines for people with MS recommend that “to achieve important fitness benefits, people with MS who have mild to moderate disability need at least 30 minutes of moderate-intensity aerobic activity 2 times per week and strength training exercises for major muscle groups 2 times per week. Meeting these guidelines may also reduce fatigue, improve mobility, and enhance elements of health-related quality of life” [16]. However, recent evidence suggests that nearly 80% of people with MS are not meeting the general public health guidelines for moderate-to-vigorous PA (ie, 30 minutes of moderate-to-vigorous PA on 5 days a week via aerobic activity and at least 2 days per week of strengthening activities, including all major muscle groups) [17,18]. Therefore, it is imperative to develop and implement evidence-based interventions that apply effective behavior change approaches to promote a physically active lifestyle by people with MS. It is important to tailor these interventions to the needs of people with MS to ensure their effectiveness at fostering long-term behavior change and positive outcomes.

Although advanced medical treatments and related rehabilitation therapies for MS have been developed, many people with MS are unable to access these health care resources due to limited mobility, fatigue, and other financial and environmental barriers [19,20]. Telerehabilitation holds substantial promise for addressing these challenges, offering a patient-centered approach that is both cost-effective and efficient. When appropriate for patients, telerehabilitation also proves advantageous and convenient for clinicians. It enables them to assess and monitor treatment responses and outcomes promptly, allowing for the optimization of intervention timing, intensity, and duration as needed [21,22]. In a study by Li et al [23], 73% of outpatients with MS reported using telehealth services with a high level of satisfaction. Additionally, a survey revealed that 93% of individuals with MS used the internet, compared with 75% of the general population [24]. Remarkably, over 80% of people with MS expressed interest in online information related to MS, including aspects like prognosis and medication, while approximately 90% were interested in online information pertaining to MS management, such as PA, diet, stress reduction, and mind-body therapies [25]. Therefore, an internet-based intervention aimed at promoting a physically active lifestyle holds great promise for many people with MS.

It is worth noting that a systematic review including 9 randomized controlled trials (RCTs) concluded that the quality of current research on telerehabilitation for people with MS is insufficient and needs to be improved [20]. The review found the interventions showed low-level evidence for reducing short-term (3 months) as well as long-term (≥6 months) disability symptoms and impairments and improving quality of life and psychological outcomes, indicating the need for studies exploring the efficacy of long-term interventions. Additionally, it is worth noting that the reviewed programs had limited data on patient experience and satisfaction, and only 3 studies were grounded in theory, particularly in social cognitive theory (SCT) for increasing PA [20]. More importantly, existing studies lacked a sound theoretical basis with behavior change researchers, and the Medical Research Council strongly advocates for a theory-based approach to complex interventions as they are effective, sustainable, and scalable [26-28]. The most commonly used theoretical framework, SCT [20,29], uses goal setting as a robust variable for participants’ increased engagement in PA and maintenance of PA 3 months after an intervention [30-35]. However, self-regulation for long-term maintenance (eg, >3 months) of PA and the psychosocial effect of managing a physically active lifestyle, such as self-efficacy, coping with challenges or relapses, and commitment, are seldom considered [36,37]. These factors are critical and contribute to maintaining control over health in the absence of a cure for MS; therefore, considering them for self-management is imperative [36-38].

Further, these SCT intervention studies did not develop an individual’s self-regulation knowledge and skills based on Bandura’s self-regulation theory [39]. It is very important for people with MS to learn self-regulation for their new behavior of PA because they could experience variations in the frequency and intensity of symptoms and impairments, especially fatigue, pain, depression, and anxiety [4,6]. As a result, individuals with MS must acquire the necessary tools to effectively regulate their engagement in PA, even when they are not feeling their best [36,37,40,41]. To maintain a physically active lifestyle over the long term, people with MS need to learn how to regulate PA engagement on top of various physical and mental challenges and to resume PA from their relapses due to different reasons (eg, flare-ups, surgery) [36,37,40,41].

Therefore, we adopted the health action process approach (HAPA) by Schwarzer et al [42] to craft our online intervention. HAPA has integrated SCT [43,44], the stages of change model [45], and the theory of planned behavior [46]. HAPA consists of two key phases: motivation phase and volition phase. In the motivation phase, an individual’s action self-efficacy, risk perception, and outcome expectancy drive the intention to change behavior. Once motivated, the volition phase involves action planning, coping planning, maintenance self-efficacy, and recovery self-efficacy, which are essential for sustaining behavior change and resuming activity after potential relapses. Additionally, recognizing and assessing an individual’s barriers and available resources are crucial for long-term behavior maintenance, as these factors influence self-efficacy across both phases of the model. To ensure the suitability of HAPA for people with MS, we validated the HAPA model and its measure battery for PA and eating behavior with people with MS [37].

In line with the community-based participatory research process, during the program development phase, we recruited community members living with MS who have successfully maintained a physically active lifestyle in accordance with the guidelines [17,18,47] for more than one year to serve as community peer leaders (henceforth called peer coaches). The peer coaches played an active role in developing and evaluating the materials for the 8 modules that constitute the intervention (see the Methods section). The peer coaches will offer invaluable peer support and assistance to the intervention group [36,48]. Moreover, we used the flipped classroom pedagogical approach to facilitate adult learning (see the Methods section).

### Objectives

The proposed study aims to assess the preliminary effectiveness of an online intervention to promote PA by people with MS (hereafter called an HAPA-MS intervention). We hypothesize that the intervention group will demonstrate (1) higher scores of HAPA components (see the Measures section) and (2) higher frequency, duration, and intensity of PA engagement immediately following the intervention and 6 months or longer after the intervention than the control group.

## Methods

The following sections provide details regarding the proposed intervention protocol, sample size calculation and power analysis, planned recruitment strategy, randomization, measures to be used for assessment, and statistical analysis, as well as adverse event and safety monitoring.

### Ethical Considerations

This study was approved by the institutional review board (IRB) for ethical and legal conduct of human subject research at the University of Illinois Urbana-Champaign. The university IRB approved the study (IRB24-0567) in expedited category 4 (collection of data through noninvasive procedures), category 6 (collection of data from voice, video, digital, or image recordings made for research purposes), and category 7 (research on individual or group characteristics or behavior or research using survey, interview, oral history, focus group, program evaluation, human factors evaluation, or quality assurance methodologies) with minimal risk. We used the following confidentiality precautions: (1) storing research data on password-protected computers or in locked cabinets in the principal investigator’s laboratory; (2) storing participant identifiers separately from the coded, participant data; and (3) for publishing, completely de-identifying the study data. There is no identification of individual participants’ data in any of the manuscript files. When a participant in the intervention group completes the 8-week program, they will be compensated with a US $220 gift card. When a participant in either the intervention group or control group completes a survey and wears an accelerometer for 7 days, they will be compensated with a US $20 gift card.

### Proposed Intervention

The intervention consists of an 8-week, online, self-regulation program, including a weekly online discussion meeting on Zoom led by peer coaches. The topics of the 8 weekly modules are as follows: (1) Introduction to the program and testing online functionalities and materials; (2) Increasing awareness of your health and lifestyle; (3) Understanding the benefits of PA; (4) Building motivation; (5) Harnessing social and environmental support; (6) Creating a personalized plan for regular PA; (7) Learning to regulate a new physically active lifestyle; and (8) Sustaining progress and planning for long-term PA. Participants will be given access to the university learning system (Canvas) to view weekly educational modules, which provide information regarding developing a physically healthy lifestyle. During the last 4 weeks, participants will progress toward learning maintenance of this newly developed physically active lifestyle. Each module is about 15 minutes in length and has built-in quizzes. The modules were developed during the first phase of the study with a patient advisory board who was actively involved in providing feedback and building the content. During each week of the study, participants will watch a module video and complete an assignment based on the topic of the week. Once they have completed these 2 steps, they will join the online discussion led by peer coaches with their group members. Each intervention group will consist of about 3 to 5 members. To familiarize participants with the online system, an onboarding session will be conducted before the intervention begins. The research team will provide online assistance throughout the 8 weeks to address any technical difficulties. Additionally, every week, participants will be provided with resources curated, evaluated, and approved by the patient advisory board and the research team concerning physically active lifestyle recommendations and research.

### Sample Size Calculation and Power Analysis

According to G*Power 3.1.9.7 [49], when we conducted a power analysis of comparing 2 groups (ie, an intervention group and a control group), an independent *t* test for estimating a sufficient sample size with a 1-tail hypothesis, at a power of 0.80, an alpha level of 0.05, and a medium-large effect size (*d*=0.65-0.70), each group will need to include 26 to 30 participants. The estimated total sample size is 52 to 60 participants. The effect size of 0.65 to 0.70 is based on the large effect sizes (*d*>0.70) of previous internet-based intervention studies [30-35]. When a condition (intervention vs control) is a between-subjects factor and time is a within-subjects factor, a power analysis of a repeated measures within-between interaction (eg, 2 conditions × 3 times) at a power of 0.80, an α level of 0.05, a medium-large effect size (*f*=0.25-0.40), and estimated 8 measures, each group will need to include 8 to 16 participants. The effect size of 0.25 to 0.40 is based on the large effect sizes (ηp^2^>0.14) of previous internet-based intervention studies [30-35]. Therefore, the total sample size of 60 (30 for the intervention group, 30 for the control group) participants is sufficient for the proposed study. In addition, using a wait-list group as a control group is recommended for developmental trials [50].

### Recruitment and Participant Selection

Recruitment will be conducted using a purposive sampling method within the United States by publishing this trial on the National Multiple Sclerosis Society website. Once participants sign up using the sign-up sheet, or by calling or emailing the research team, the team will conduct a short telephonic interview, which involves screening the participants based on the inclusion criteria (see Textbox 1). Once participants are recruited, they will be asked to fill out the baseline survey and wear an accelerometer for 7 days. The baseline assessment materials will be sent to the participant via physical mail, with a prepaid postage packet provided to send the device back to the research lab. We will recruit 60 participants with any type of MS who satisfy the inclusion criteria (see Textbox 1).

**Inclusion and exclusion criteria for participant enrollment into the trial.**

**Inclusion criteria**

- ≥18 years old and
- Any type of multiple sclerosis
- Not maintained 30 minutes of moderate-to-vigorous physical activity (PA) per day for 2 days of the week during the previous 6 months
- Relapse-free for the past 30 days
- Have a low risk of contraindications of PA indicated by no more than a single “yes” response on the Physical Activity Readiness Questionnaire (PAR-Q) [51]
- Able to walk with or without an assistive device (ie, Patient-Determined Disease Steps [PDDS] score from 0 to 6) [52]
- Be willing to be randomized to an intervention or control group, complete the surveys and questionnaires, and wear an accelerometer during the intervention period
- Have reliable internet access

**Exclusion criteria**

- Godin Leisure-Time Exercise Questionnaire (GLTEQ) score >14 [53]
- No computer nor mobile device access to the internet
- PDDS score=7 (ie, using a wheelchair or scooter most of the time) or PDDS score=8 (being bedridden)
- Anyone living outside of the United States
- Anyone who is unable to speak, read, and write in English
- Not willing to be randomized to an intervention or control group, complete the surveys and questionnaires, and wear an accelerometer during the intervention period
- No access to reliable internet

### Randomization

**Figure 1:**
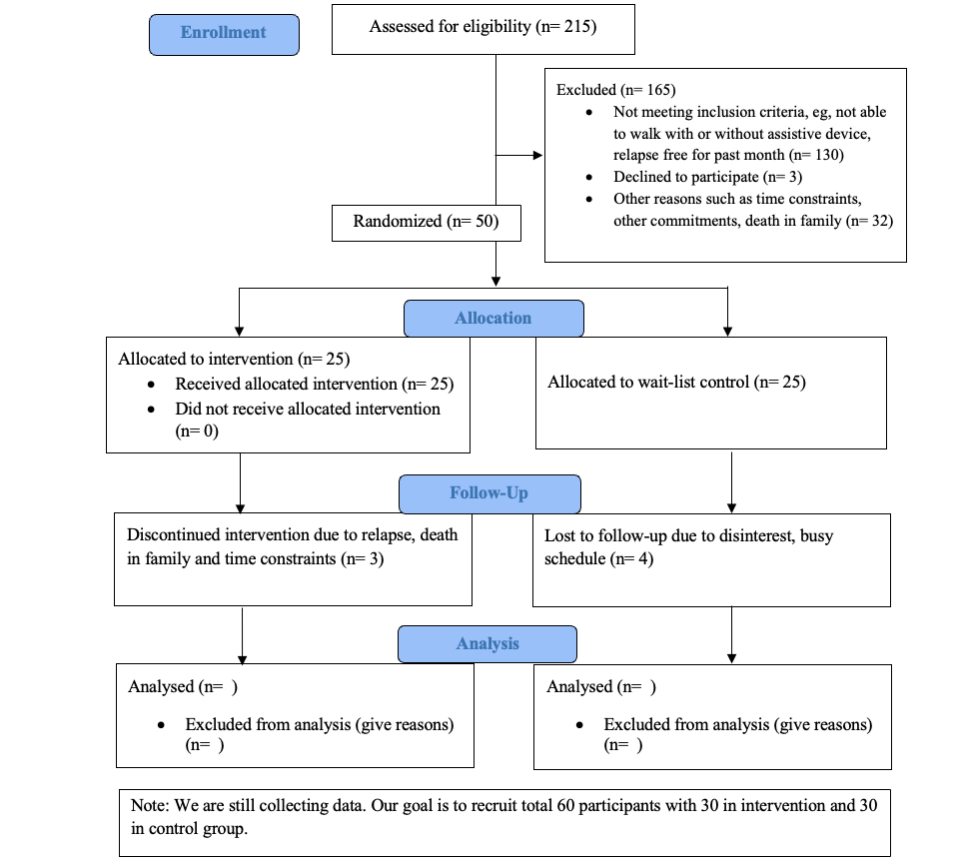
CONSORT (Consolidated Standards of Reporting Trials) flow diagram for the health action process approach-multiple sclerosis (HAPA-MS) randomized controlled trial.

Research assistants enroll participants prior to randomization. The research team, including the principal investigator and a research assistant, will conduct matched, paired randomization based on Patient-Determined Disease Steps (PDDS) scores. Randomization will be conducted by flipping a coin until there are no significant differences in PDDS, Godin Leisure-Time Exercise Questionnaire (GLTEQ), age, education levels, income, and gender. To assign qualified participants in a timely manner, we will randomize every 8 to 10 participants into the intervention or wait-list control group (Figure 1). Participants will be notified regarding their allocation via email and phone call.

### Data Collection

**Figure 2:**
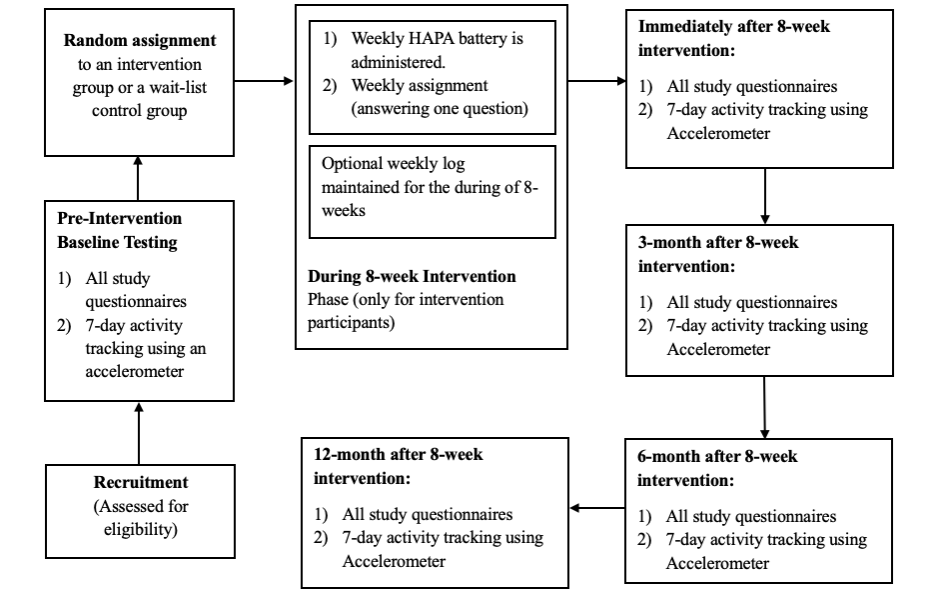
A flow diagram of data collection time points for the health action process approach-multiple sclerosis (HAPA-MS) randomized controlled trial.

Study assessments will be conducted at baseline, postintervention (ie, after the 8-week intervention), 3 months postintervention, 6 months postintervention, and an optional follow-up at 12 months postintervention (Figure 2). Throughout the testing phase, participants will be provided with prepaid posting labels and packages for easy access and hassle-free delivery of study material.

#### Baseline Demographics

During the screening process, basic demographic information will be collected, including age, gender, sex, ethnicity, level of education, income, and employment status. We will also collect additional information, including the type of MS, MS diagnostic tests, MS diagnosis year, and availability of PA-related resources around the individual. We will use this information to further enhance our knowledge regarding factors that may be related to supporting a physically active lifestyle.

#### PA Measures

ActiGraph (wGT3X-BT; ActiGraph LLC) accelerometers will be used to collect PA data during the data collection time points. Participants will be asked to wear the accelerometer around their waist on the nondominant side for 7 days and complete the accelerometer log sheet, including time stamps of wear time. ActiGraph accelerometers were deemed fit for collecting step count data. One study has shown that ActiGraph accelerometers accurately measure steps during moderate and fast walking speeds by both people with MS and control groups. Motl et al [54] indicated a slight underestimation of step count during slow-paced walking using an accelerometer with both people with MS and control groups. Given this, this study will encourage the intervention group participants to keep an optional PA log from the start of the intervention until the end of the study. The log includes recording information on the type, duration, frequency, and intensity of activities performed throughout the day.

#### Questionnaire Measures

The study will use the questionnaires and measures described in the following paragraphs to collect data regarding components of HAPA, PA, functionality, pain, fatigue, anxiety, and depression.

The Abbreviated Late Life Function and Disability Inventory has 2 subscales: function and disability. It has 15 items about the difficulty and frequency of performing physical activities rated on a 5-point Likert scale. Cronbach α values are 0.58 to 0.85 [55].

The McGill Pain Questionnaire has a 15-item adjective checklist that captures sensory and affective dimensions of pain experienced during the past 4 weeks. The items are rated on a 4-point scale [56]. Its Cronbach α value is 0.88 [57].

The Fatigue Scale for Motor and Cognitive Functions has 20 items (10 for cognitive and 10 for physical fatigue) rated on a 5-point scale. Its Cronbach α values are 0.91 to 0.95. Cutoff values are available to classify patients as mildly, moderately, or severely fatigued [58].

The Hospital Anxiety and Depression Scale has 14 items (7 for anxiety and 7 for depression) rated on a 4-point scale. The depression (*r*=0.70, *P*<.001) and anxiety (*r*=0.74, *P*<.001) subscales are correlated with psychiatric ratings [59]. Cronbach α values are 0.82 to 0.83 [57].

The HAPA battery of risk perception includes the subscales of Disease Threatening, Exam Result Threatening, Risk to Self, Risk to Others, Relative Vulnerability, and Perceived Severity. Each of these subscales has 10 to 13 items based on secondary health conditions in people with MS that are rated on a 7-point scale. Cronbach α values are 0.92 to 0.94 [37]. The HAPA battery of exercise benefits includes the subscales of Exercise Influence on Health Problems (13 items) and Outcome Expectancy (14 items). Cronbach α values are 0.79 to 0.93 [37]. The HAPA battery of motivation includes the subscales of Intention to Do PA (8 items) and Motivational Self-Efficacy (3 items). Cronbach α values are 0.90 and 0.85, respectively [37]. The Cognitive and Behavioral Process of Change Scale has 30 items (15 for the cognitive subscale and 15 for the behavioral subscale) rated on a 5-point scale. Cronbach α values are 0.67 to 0.86 [60]. The Exercise Goal Setting and Planning Scale has 20 items (10 for the goal setting subscale and 10 for the planning subscale) rated on a 5-point scale. Cronbach α values are 0.89 and 0.87, respectively [61]. The Barriers to Health Promotion Activities Scale has 18 items rated on a 4-point scale. Its Cronbach α value is 0.82 [62]. The PA-specific Social Provisions Scale has 24 items rated on a 4-point scale. Its Cronbach α value is 0.89 [57,63]. The Physical Activity Self-Regulation Scale has 12 items rated on a 5-point scale. Cronbach α values are 0.79 to 0.94 [64]. The HAPA battery of PA volition includes the subscales of PA Action Planning (5 items), PA Coping Planning (4 items), Recovery Self-Efficacy (3 items), and Coping Self-Efficacy (13 items) rated on a 4-point scale. Cronbach α values are 0.91 to 0.97 [37].

PA and sedentary behavior will also be assessed using patient-reported scales, that is, the Physical Activity Scale for Individuals with Physical Disabilities (PASIPD) [65] and GLTEQ. Cronbach α values for the PASIPD are 0.37 to 0.65 and for the GLTEQ are 0.46 to 0.94 [65-67].

### Statistical Analysis

Research assistants will monitor survey completion and accelerometer data sufficiency. When participants return their survey and accelerometers, research assistants will check whether the participants have not responded to the entire survey. If there are missing responses, research assistants will email or call the participants to encourage them to respond to the missing items. The participants will be reminded twice, once each at 2 and 4 weeks, after they return the survey. If their accelerometers do not record valid data, research assistants will contact the participants and ask if they are willing to wear it for 7 days. After those attempts, when we curate the data, we will code missing data as 999.

Our analytic approach will use an intention-to-treat framework, and mixed models will yield unbiased results if the data are “missing at random” [68]. Additional sensitivity analyses will be performed to test for informative dropouts [68]. We will apply pattern-mixture models by stratifying our data by dropout patterns and fitting separate regression models to strata [68]. SPSS 26 (IBM Corp) will be used to conduct descriptive analyses (means, standard deviations), chi-squared tests, independent *t* tests, paired *t* tests, repeated measures ANOVAs, and 2-way (any 2 factors of conditions, disability severity levels, and time) and 3-way (conditions × disability severity levels × time) mixed model ANOVAs. Conditions and disability severity levels are between-subjects factors. Time is a within-subjects factor. We will use 2-way and 3-way ANOVAs to compare measured variables of the intervention and control groups. The significance level is set at .05. We will examine effect sizes of partial ηp2 (0.01, 0.06, and 0.14 as small, moderate, and large effect sizes, respectively) and Cohen *d* (0.20, 0.50, and 0.80 as small, moderate, and large effect sizes, respectively) [69]. We hypothesize that the intervention group will have stronger motivation and self-regulation, more PA, and better health conditions than the control group.

### Adverse Event and Safety Monitoring

Regarding safety monitoring, the Physical Activity Readiness Questionnaire [51] will be used as a measure to check for any comorbidities or doctors’ suggestions for PA. If an individual answers more than one “yes” on this questionnaire, they will not be recruited. If they answer with one “yes,” we ask them to consult their doctor about whether it is fine for them to join the study. We periodically ask participants about their regular MS testing and infusion schedules to avoid fatigue or inconvenience. Participants are also encouraged to keep a note of exacerbations or flare-ups. The IRB recognizes this clinical trial as “minimal risk.” For data safety and monitoring, deidentified data will be stored in a secured database (a HIPAA-compliant Box folder only accessible to IRB-approved team members). The research team will conduct routine checks on saved data.

## Results

The study was funded in April 2018. We conducted peer coach selection interviews from July 2018 through October 2018. Peer coaches codeveloped and evaluated each module of the online program from November 2018 through January 2020. We iteratively revised the modules based on the peer coaches’ feedback. During the pandemic, participants preferred to discontinue the study. The modules also had to be migrated from the Moodle system to a new online learning system, Canvas, in 2022 due to changes in the university’s online learning system.

We invited peer coaches to review the accessibility of online modules in fall 2022. We finalized the module accessibility and trained peer coaches on how to use the online modules in December 2022. Since February 2023, we have recruited 50 participants to the trial who have been assigned to the intervention group (n=25) or control group (n=25). During the follow-up stage, 3 participants dropped out of the intervention group, and 4 participants dropped out of the control group. Enrollment ended in 2024, and data collection is expected to conclude by December 2025, with results anticipated to be published in January 2026.

## Discussion

### Overview

This protocol outlines an RCT to assess the preliminary effectiveness of an online program designed to promote a physically active lifestyle among individuals with MS, utilizing a community-based participatory approach [70]. We expect that the intervention group will be more physically active, especially 6 months after graduating from the program, and will have more phase-specific self-efficacy than the control group. Compared with prior work applying SCT [20,30-35], we used a hybrid framework, HAPA, which provides a comprehensive approach considering an individual’s cognitive processing (ie, different phase-specific self-efficacy), behavior implementation (ie, action and coping planning), and living circumstances (ie, barriers, facilitators, or supportive resources). Peer coaches will lead discussions regarding where and how to gain motivation, what and how to practice self-selected PA and self-set goals each week, and how to cope with barriers and find countable resources to gradually adopt a physically active lifestyle over the program period week by week. We expect that PA logs from the intervention group will show their individualized PA profiles regarding activity types, frequency, and duration.

Additionally, we will evaluate the program’s capacity to sustain these new lifestyle changes over an extended period, specifically beyond 3 months. To our knowledge, this is one of a few studies implementing a flipped classroom intervention customized to the unique needs and preferences of adults with MS. The adoption of a flipped classroom design, along with peer coaches, not only fosters deep learning experiences but also amplifies interaction and facilitates vicarious learning from the experiences and knowledge of others [71]. This approach empowers participants to take an active role in their learning journey, promoting self-directed and autonomous learning. Additionally, it significantly enhances motivation, thereby cultivating an individual’s interest and capacity for self-regulation. A hybrid theoretical model rooted in evidence-based principles was used to construct the program’s structure and modules. These foundations have been drawn from the research team’s prior research endeavors [36,37,41]. Of equal significance, we have effectively translated these theoretical components into each intervention module, considering the perspectives of individuals living with MS. Using an iterative process to revise each module and collaborating closely with peer coaches helped us ensure that the intervention effectively addresses the specific needs and requirements of the target community. This iterative methodology proved invaluable in our collaboration with community researchers [72]. They provided us with crucial, patient-centered feedback, enabling us to enhance the content of the 8-week modules. It is important to recognize that this process is far from a mechanical repetition; rather, it embodies a deep reflective approach that aids in the development of information tailored to the unique needs of individuals living with MS. Furthermore, our adoption of community-based participatory research not only facilitates the seamless implementation of the program within the community but also enhances its overall applicability. Peer coaches play a vital role in enhancing multiple phases of self-efficacy among members of the intervention group [39,44]. They serve as role models for participants, showcasing a range of successful experiences in sustaining a PA routine while also delivering positive and empathetic feedback to their fellow participants. These actions constitute the primary mechanisms for bolstering an individual’s self-efficacy [39,44]. Moreover, the peer coach’s wealth of experience and expertise in this context is instrumental for fostering connections and personalizing the program further. The practices embedded within the weekly modules will facilitate participants in discovering physical activities that are both feasible and enjoyable, paving the way for the development of a sustainable lifestyle. Research has consistently emphasized the significance of finding enjoyment in sustaining a long-term active lifestyle [73]. Finally, we intend to implement the program using a telerehabilitation method. Considering that a substantial number of people with MS have already used the internet and mobile devices to access health information and resources [24], this online approach holds the promise of being a more effective and valid means to enhance the health and well-being of people with MS.

Although the HAPA-MS program promises much, we are aware of some limitations. For example, when we intend to recruit people with mild to severe mobility disabilities, future studies should aim to build websites that are more accessibility friendly, particularly for people with functional limitations in dexterity and deafness. Wearing the current type of accelerometer may not be as convenient as a smartwatch with an accelerometer feature. This will reduce compliance with the accelerometer wearing protocol. Although we designed a comprehensive survey composed of the HAPA components and other psychosocial factors, the survey will take about an hour to complete. It may increase the challenges with managing missing responses, especially during the follow-ups. Moreover, future studies should consider random sampling rather than convenience and purposive sampling, enhancing the robustness of the trial.

### Conclusion

In addition to the advancement of scientific literature on health promotion, the results of the proposed RCT could have a significant impact on activity and lifestyle recommendations for people with MS. Our study will determine the preliminary effectiveness of using a HAPA-based online intervention to promote an active lifestyle by people with MS. Ultimately, this approach can contribute significantly to the self-regulation of PA behavior over the long term and has the potential for widespread acceptance among many other individuals living with MS.

## Appendix

Multimedia Appendix 1 SPIRIT checklist.

## Abbreviations

GLTEQ: Godin Leisure-Time Exercise Questionnaire
HAPA: health action process approach
IRB: institutional review board
MS: multiple sclerosis
PA: physical activity
PASIPD: Physical Activity Scale for Individuals With Physical Disabilities
PDDS: Patient-Determined Disease Steps
RCT: randomized controlled trial
SCT: social cognitive theory

## Data Availability

The data sets generated or analyzed during this study are available from the corresponding author on reasonable request.
